# Reply to Comment on Tsai, Y.-C., et al. Association of Stress-Induced Hyperglycemia and Diabetic Hyperglycemia with Mortality in Patients with Traumatic Brain Injury: Analysis of a Propensity Score-Matched Population. *Int. J. Environ. Res. Public Health* 2020, *17*, 4266

**DOI:** 10.3390/ijerph18052531

**Published:** 2021-03-04

**Authors:** Yu-Chin Tsai, Shao-Chun Wu, Ting-Min Hsieh, Hang-Tsung Liu, Chun-Ying Huang, Sheng-En Chou, Wei-Ti Su, Shiun-Yuan Hsu, Ching-Hua Hsieh

**Affiliations:** 1 Department of Neurosurgery, Kaohsiung Chang Gung Memorial Hospital and Chang Gung University College of Medicine, Kaohsiung 83301, Taiwan; vagility@gmail.com; 2 Department of Anesthesiology, Kaohsiung Chang Gung Memorial Hospital and Chang Gung University College of Medicine, Kaohsiung 83301, Taiwan; shaochunwu@gmail.com; 3 Department of Trauma Surgery, Kaohsiung Chang Gung Memorial Hospital and Chang Gung University College of Medicine, Kaohsiung 83301, Taiwan; hs168hs168@gmail.com (T.-M.H.); htl1688@yahoo.com.tw (H.-T.L.); junyinhaung@yahoo.com.tw (C.-Y.H.); athenechou@gmail.com (S.-E.C.); s101132@adm.cgmh.org.tw (W.-T.S.); ah.lucy@hotmail.com (S.-Y.H.)

Thank you for Eduardo Mekitarian Filho’s appreciation of our work on the study of stress-induced hyperglycemia (SIH) and diabetic hyperglycemia (DH) in patients with traumatic brain injuries. According to our previous studies on SIH and DH in patients with various illnesses or other conditions [1,2,3,4,5,6,7,8], the mortality rate is always worse for patients with SIH than for patients with nondiabetic normoglycemia; however, such poor outcomes are not always observed in those with DH. Differentiating between the etiologies of a patient’s hyperglycemic status is important not only because they can affect patient outcome but also because the goals for glucose control differ between SIH and DH. Aggressive glucose control (i.e., maintaining glucose levels within the normal range) may be limited for those with DH but not for those with SIH; because although some studies reported conflicting results, SIH is believed to be a physiological response that parallels the elevation of catecholamines in the body in its attempt to cope with major stress. Moreover, many questions remain unanswered in this field. For example, why do some patients develop SIH while others do not? What is the magnitude of stress required for the development of SIH? What are the risk factors for SIH? Do genetic or epigenetic factors play a role in the occurrence of SIH? What is the goal of glucose control in patients with SIH? Do any treatments specifically benefit patients with SIH? What is the role of steroids in the management of SIH? As diabetic patients may also face major stress, what is the effect of SIH in those with diabetes [4]? There are still many unanswered questions and unexplored areas with regard to SIH; thus, further research is required to solve this problem.
